# The Hospitals Association

**Published:** 1904-02-27

**Authors:** 


### HOSPITAL ADMINISTRATION.

### CONSTRUCTION AND ECONOMICS.

### THE HOSPITALS ASSOCIATION.

LONDON'S AMBULANCE ORGANISATION.

The annual meeting of the Hospitals Association was held on the 17th inst. in the Reference Library of the Association, The Hospital Building, Southampton Street, Strand. Mr. Thomas Bryant, F.R.C.S., the President, took the chair.

The Hon. Secretary, Mr. Sydney Phillips, B.A., Steward of St. Thomas's Hospital, read the report of the Council, which stated:—

During the past year three general meetings have been held. They were all well attended, and useful discussions took place on each occasion. At the meetings in March and December, Sir Henry Burdett read papers on the question now so much to the front, as to the desirability of moving certain hospitals from their present sites to districts where hospital accommodation is deficient. It is a matter for congratulation that since the reading of the first paper one hospital (King's College) has decided to move to South London, and it is to be hoped that the scheme will be widely supported by the charitable public.

At the October meeting a discussion was opened by Dr. Savill (late superintendent of Paddington Infirmary) and Miss Gibson (matron of Birmingham Infirmary), on the "Report of the Departmental Committee on Nursing appointed by the President of the Local Government Board," and the following resolution was carried *nem. con.*:—

"That this meeting disagrees with the proposals of the Departmental Committee on Nursing to create Minor Training Schools in small Workhouses, and with the recommendation that a probationer who has undergone at least one year's training in such schools should be considered and called a 'qualified nurse.' This meeting believes that the adoption of the recommendations would have a most detrimental effect upon the position and character of nursing as a profession and an equally injurious influence on the Poor-law Service and public interests. It would ask the President of the Local Government Board to receive a deputation on the subject."

The President of the Local Government Board (the Right Hon. Walter Long, M.P.), in acknowledging a copy of the resolution, said he would "be glad to give the matter his careful attention, but that he did not think it necessary to trouble a deputation to attend."

The Council have to tender their grateful thanks to the treasurer of St. Thomas's Hospital and to the council of Charing Cross Hospital for their kindness in lending their board-rooms for the meetings of the Association.

The Council regret that the reference library has not been more extensively used during the past year. A very useful collection of books on all branches of hospital administration is at the disposal of members, in a very accessible spot within a few yards of Charing Cross, and should prove of inestimable advantage to hospital officials.

The Council regret to record the deaths of two of their number, Mr. G. B. Lloyd and the Rev. Prebendary Kitto. Mr. Lloyd was one of the original members of the provisional committee at the foundation of the Association in 1884, and although circumstances had of late prevented him from taking any active part in the work of the Association, he was strongly in sympathy with its aims. The Rev. Prebendary Kitto joined the Council in 1897, and his loss will be much felt.

Mr. Lancelot Hugh Smith, representing the Home Hospitals Association, has been elected to fill one of the vacant seats.

Mr. L. S. Bristowe has resigned the office of Treasurer, in consequence of his appointment to a Judgeship in the Transvaal. The Council desire to record their deep sense of the value of Mr. Bristowe's services to the Association. To him is due the passing of the "Mortmain and Charitable Uses Act, 1891," which has conferred so great a boon on hospitals and other charities by removing the legal restrictions in respect to gifts by will under which these institutions had so long suffered.

Mr. Conrad W. Thies, Secretary of the Royal Free Hospital, has been appointed to the vacant post.

Sir Savile Crossley's term of office as President having expired, Mr. Thomas Bryant, Consulting Surgeon of Guy's Hospital, has kindly consented to accept the office. The Council thanks Sir Savile Crossley for his valuable services in the chair during the past two years, and at the same time they warmly welcome his successor. Mr. Bryant has already very kindly presided at two of the general meetings, and the Council are sure that his occupancy of the presidential chair will be of great advantage to the Association.

The Council regret that no further progress has been made in the movement for freeing hospitals from local rates. They hope that the Central Hospital Council, which has taken charge of the matter, will still press forward with their bill in the ensuing session of Parliament.

The Council desire to point out that a considerable increase in membership is necessary if the Association is to continue active work on its present lines. A neutral platform for the discussion of hospital matters is surely an absolute necessity for so important a department of civic life as hospitals are, and this Association has for the past 20 years supplied that platform. Much good has resulted, and the Council therefore appeal to all who are interested in hospital work to enrol themselves as members of the Association.

The balance-sheet annexed to the report showed annual subscriptions of £38 and an expenditure of £55 for the past year, with funds in hand of £711.

Mr. Thomas Ryan, the hon. secretary of the Street Ambulance Branch of the Association—the "Bischoffsheim" Ambulance Service of London—then read the report of the committee of the branch. This showed that since 1891 an average of about 2,000 cases per annum had been attended to, the number of times the ambulances had been employed totalling over 22,000. Continuing the report stated:

The extent of the work that has been done is highly satisfactory, and the Committee feel that the fact that London is indebted for it to the public spirit and munificence of one person, Mr. H. L. Bischoffsheim, is one that calls for grateful recognition. It will be remembered that Mr. Bischoffsheim provided nearly the whole of the necessary funds for the establishment of the Service, and the maintenance charges, about £350 a year, have been borne by him ever since. The committee know that it is a great satisfaction to Mr. Bischoffsheim to feel that his liberality, which he would be the last to wish mentioned, has been productive of so much good. No one who has seen a bad case of street accident conveyed to hospital in a cab can doubt for a moment that the ambulances have been the means of saving many lives and of preventing a great deal of suffering.

The number of stations in London is 55 of which 25 are in thoroughfares, 15 at hospitals, 9 at fire brigade stations, and 6 at public buildings and elsewhere.

The Chairman moved the adoption of both reports presented, expressing especial satisfaction with the second. He thought that all must feel deeply grateful to Mr. Bischoffsheim, and he suggested that a letter should be addressed to him conveying an expression of the thanks of that meeting. Too much could not be said for the good work done.

Sir Henry Burdett seconded the resolution. With reference to the question of street ambulances he said it was very remarkable that the recent discussion in the columns of the *Times*, followed by a leading article, should have practically ignored Mr. Bischoffsheim's work altogether. It did not seem to be recognised that all that had been done hitherto had been by that gentleman's agency. In 1889 Mr. Bischoffsheim had his attention drawn to the matter. He said, if a system could be organised he would be prepared to pay the cost of the equipment and of the working, and since that time he had provided many thousands of pounds. Some 22,000 cases had been dealt with, and in many instances saved from great injury and suffering by the working of the Bischoffsheim system. Twice during its existence the Association had approached the London County Council on the subject, and each time the Council had appointed a committee and issued a report. The last of these, dated December 5th, 1902, contained a fairly full account of the work done in London and in Continental and American cities, and showed plainly that London was far behind every other great city of the world in this matter. What did that mean to the inhabitants? It meant that in a densely-populated city there must be many cases of sudden illness and accident improperly handled—that simple fractures were converted into compound and complicated fractures; that an ordinary lesion which if handled from the first with knowledge, and conveyed on an ambulance to a hospital would probably mean rest and treatment for a few weeks, might have the most serious consequences, and even the loss of limb or of life. Why was it that London had no adequate street ambulance service? Mr. Bischoffsheim had not only provided money and done all that a private citizen could do, but he had repeatedly pointed out that the limit of what he could do had been reached because he could not get sites for more ambulance stations from those who by law were in control of public spaces and other suitable situations. Now they were on the eve of the elections for the County Council. They were face to face with the fact that in a matter of public importance, when a private citizen had done his best the inertness of the municipal authorities stood out prominently. His (Sir Henry's) proposal was that electors in every division of the Metropolis should ask their candidates what attitude they would take up in regard to the street ambulance question if elected. No doubt a number of the present members desired to have a proper service, but the inertness of the majority had caused a deadlock, and so nothing had been done—and nothing would be done unless Londoners roused themselves and brought pressure to bear. This was not a question of finance. An adequate service of street ambulances for the whole of the Metropolis could be provided for a sum so small that it could have no effect on the rates. It had been suggested that the Metropolitan Asylums Board which already was responsible for the removal of infectious cases, should undertake the work, but that body had not the necessary statutory powers to do so efficiently, and unless the London County Council, which had charge of the necessary sites, accepted the responsibility it would never be adequately discharged.

### Horse and Motor Ambulances.

As to the methods that should be adopted, there appeared to be a great deal of misapprehension. People seemed to consider there should be motor and horse ambulances provided. No doubt a few of these might be tried, but they would entail a very heavy and constant expense because, if such a service was to be kept efficient, they must have a medical service in connection with it which would entail a large annual outlay for salaries. It had been suggested that the large hospitals might co-operate and tell off some of their senior students on a rota for the purpose. But the deans of the medical schools declared that they had no men they could spare; that in fact the medical curriculum was so exacting and ward work in the hospitals was so heavy that the students were even now not numerous enough to overtake it, and much of it had to be done by the nurses in consequence. Then as to the practical question, it was generally better for the patient to be moved on to a hand litter on the level of the pavement immediately than to wait for the arrival of a horse ambulance and be lifted into it and driven away. He (Sir Henry) believed the best plan would be to continue the horse and hand litter service, so that the police or whoever took out the hand litter from the cab stand or fire station could ring a bell for the faster vehicle. It should be understood that cases from each point should be taken to a certain hospital along an agreed-upon route so that they could be met on the way. Mr. Bischoffsheim had all along said he was prepared to provide either horse or motor ambulances if it could be shown that they were required and that proper accommodation would be provided. It was due to Mr. Bischoffsheim that this should clearly be brought out, because circulars and letters had been issued which seemed to imply that nothing had been done; and the fact that Mr. Bischoffsheim had kept in the background had led people to make statements which might mislead the press and the public. He (Sir Henry) made bold to say that if they could get the matter before the County Council candidates so as to have their views expressed on the subject, that London would have an adequate and efficient street ambulance service thoroughly organised and equipped within a couple of years. They all knew that when the County Council put its back into anything it was done very well; and if the public would only show a modicum of interest in this question within the next few weeks, London would soon take its proper place in this respect among the cities of the world. At Liverpool, Birkenhead, Manchester, Newcastle, Huddersfield, Bolton, Burnley, Hull, Sheffield, Leeds, and Wolverhampton they had such a service, and why they in London should go on letting their citizens suffer unavoidable risks and pain he could not understand.

Turning to other matters mentioned in the report of the Association Sir Henry thought they should not let the occasion pass without a word in reference to the deaths of their colleagues on the council, Mr. Lloyd and Prebendary Kitto. Mr. Lloyd it was who took up the question of hospital accounts, and led to a great improvement in that respect. In him they had lost not only a loyal friend but one whose services had been of great value to charitable institutions. Than Prebendary Kitto he knew of none with a more generous heart or who took a wider view of hospital questions. In conclusion, he thanked the meeting for the letter which was to be sent to Mr. Bischoffsheim, to whom London owed a great debt of gratitude.

The officers and retiring members of the council were re-elected, and a vote of thanks to them and to the chairman was moved by Sir Henry Burdett, who congratulated Mr. Bryant on a year of office which had been productive of a movement of real benefit to the hospital world. They had been able to show that while on the whole the hospitals of

London were so placed as to be of the greatest benefit, yet some would be of more service in other localities. In two of these cases the hospital authorities had taken up the matter. King's had decided to remove to Camberwell, while at St. George's the question was under the consideration of a committee appointed about a month after the meeting at the Caxton Hall. Then, as a result of the discussion at Charing Cross, St. Bartholomew's had decided to enlarge their scheme, and when the Post Office came in, and by taking the land prevented this, they had decided to move away some of the buildings so as to enable the hospital to be built of adequate size for the 700 beds required. Next to having an enlarged site the best thing was to remove the nursing home and medical college. The Post Office had agreed to make a 40-foot road on their site closely adjoining St. Bartholomew's, and further to co-operate with the architects of the hospital so as to give it the maximum of light and air. They had thus the hope that the new St. Bartholomew's would be worthy of its name and traditions.

Mr. Ryan seconded the resolution, which was carried, and Mr. Bryant in acknowledging it said he was quite sure all those who were present at the Charing Cross meeting would acknowledge that Sir Henry Burdett had done a very great deal for London and St. Bartholomew's in particular by bringing the question to the position it now occupied. He did not think it would have progressed so far unless Sir Henry had given it such a push that the public could not shut their eyes to what he urged, and so he told his friends at St. Bart's.

The meeting then separated.

